# Multi-year school-based implementation and student outcomes of an evidence-based risk reduction intervention

**DOI:** 10.1186/s13012-016-0539-7

**Published:** 2017-02-10

**Authors:** Bo Wang, Bonita Stanton, Lynette Deveaux, Sonja Lunn, Glenda Rolle, Richard Adderley, Maxwell Poitier, Veronica Koci, Sharon Marshall, Perry Gomez

**Affiliations:** 1 Division of Behavioral Sciences, Department of Family Medicine and Public Health Sciences, Wayne State University School of Medicine, 6135 Woodward Ave, Detroit, MI 48202 USA; 2 Seton Hall-Hackensack Meridian School of Medicine, Hackensack Meridian Health, 400 South Orange Avenue, South Orange, NJ 07079 USA; 3 Office of HIV/AIDS, Ministry of Health, Shirley Street, Nassau, Bahamas; 4 Ministry of Education, Thompson Boulevard, PO Box N-3913, Nassau, Bahamas; 5 Department of Pediatrics, Division of Adolescent Medicine, Wayne State University School of Medicine, Children’s Hospital of Michigan, 3901 Beaubien Street, Detroit, MI48201 USA

**Keywords:** Pattern of implementation, Booster session, HIV prevention, Program provider, Program outcomes, Adolescents, Program evaluation

## Abstract

**Background:**

Intervention effects observed in efficacy trials are rarely replicated when the interventions are broadly disseminated, underscoring the need for more information about factors influencing real-life implementation and program impact. Using data from the ongoing national implementation of an evidence-based HIV prevention program [Focus on Youth in The Caribbean (FOYC)] in The Bahamas, this study examines factors influencing teachers’ patterns of implementation, the impact of teachers’ initial implementation of FOYC, and subsequent delivery of the booster sessions on students’ outcomes.

**Methods:**

Data were collected from the 80 government elementary and 34 middle schools between 2011 and 2014, involving 208 grade 6, 75 grade 7, and 58 grade 8 teachers and 4411 students initially in grade 6 and followed for 3 years. Student outcomes include HIV/AIDS knowledge, reproductive health skills, self-efficacy, and intention to use protection. Data from teachers includes implementation and modification of the curriculum, attitudes towards the prevention program, comfort level with the curriculum, and attendance at training workshops. Structural equation modeling and mixed-effect modeling analyses were applied to examine the impact of teachers’ implementation.

**Results:**

Teachers’ attitudes towards and comfort with the intervention curriculum, and attendance at the curriculum training workshop had a direct effect on teachers’ patterns of implementation, which had a direct effect on student outcomes. Teachers’ attitudes had a direct positive effect on student outcomes. Teachers’ training in interactive teaching methods and longer duration as teachers were positively associated with teachers’ comfort with the curriculum. High-quality implementation in grade 6 was significantly related to student outcomes in grades 6 and 7 post-implementation. Level of implementation of the booster sessions in grades 7 and 8 were likewise significantly related to subsequent student outcomes in both grades.

**Conclusions:**

High-quality initial implementation of a prevention program is significantly related to better program outcomes. Poor subsequent delivery of booster sessions can undermine the positive effects from the initial implementation while strong subsequent delivery of booster sessions can partially overcome poor initial implementation.

**Electronic supplementary material:**

The online version of this article (doi:10.1186/s13012-016-0539-7) contains supplementary material, which is available to authorized users.

## Background

The field of HIV prevention is maturing from a primary focus on intervention development to one that includes development and dissemination and implementation of effective interventions [1]. The many challenges encountered in implementation in real world settings result in a wide range of changes to the original intervention design (e.g., selectively implementing program components, dropping core elements, substantially altering the design) and/or implementation delivery quality issues which impair program outcomes [2–5]. Prevention programs implemented in schools outside of efficacy trials are typically not implemented with high quality [6].

Numerous studies across many fields have examined factors influencing implementation of school-based prevention programs and identified a range of factors which appear to facilitate or impede teacher implementation efforts: teacher training, program characteristics, teacher characteristics, and organizational characteristics [7–9]. Teacher/program provider training is well recognized as an essential component of successful implementation of prevention curricula [10, 11]. A number of teacher characteristics have been found to predict the fidelity of implementation including teacher’s favorable attitude towards prevention programs [12], confidence in their ability to teach prevention program and to affect student performance [13], perception of community ownership of the program [14], and shorter duration of time as teacher [15]. Organizational characteristics including receptivity to the prevention program, support by the principal or other school administrators, and the organization’s readiness to implement new programs have been shown to be related to fidelity of implementation [7, 12]. Students’ engagement in the intervention curriculum appears to promote teacher’s implementation fidelity [16]. While there is increasing recognition about specific teacher- and school-level factors that influence implementation of prevention programs, little is known about how these factors interact and the complex ways in which teachers’ attitudes and self-efficacy are influenced by education, training, and school administrators’ support, and the extent to which attitudes and self-efficacy influence program implementation and outcomes. In this manuscript, “self-efficacy” refers to teacher’s beliefs about his/her ability to implement the intervention curriculum; teacher’s “confidence” is a more general measure of teacher’s belief in the inherent value of the intervention curriculum if it is delivered appropriately. Teacher’s confidence leads to self-efficacy.

Numerous implementation models, theories and frameworks have been proposed to summarize factors at multiple levels of the social organizational context that potentially influence the process of the translating research-based effective intervention into practice [17–19]. The exploration, preparation, implementation and sustainment (EPIS) framework provides a comprehensive conceptual model to guide implementation research across the dissemination-implementation-sustainment spectrum. The EPIS model articulates variables that may play crucial roles at different phases in the implementation process and their impact on the ultimate success of intervention delivery. The model examines both the ideological and structural fit of the wider environment (the “outer context” elements) as well as the local culture and climate and providers’ characteristics and attitudes (the “inner context” elements) with regard to the intervention as indicators or guides to moving the intervention towards successful implementation [20]. EPIS emphasizes the significant role of sustained leadership and ownership over new interventions, and ongoing support and incentives for implementation as key to successful implementation and long-term sustainability. In the present study assessing the active implementation phase of school-based delivery of an evidence-based sexual risk-reduction intervention, we focus on the role of one subset of “inner context” factors, the “Individual adopter (i.e., teacher) characteristics”. Specifically, we seek to understand the relationships among the individual adopter characteristics and how they influence the implementation of the FOYC intervention. Our hypothesized conceptual model was developed based on the empirical literature [21] and the EPIS framework with a focus on the active implementation phase and “inner context” factors. We hypothesize that teachers’ attitudes towards the intervention and their comfort with the curriculum have a direct positive effect on implementation, which in turn affects student outcomes. Teacher’s comfort is influenced by their teaching experience, training, and student engagement. Teachers’ initial implementation and subsequent delivery of booster sessions have a direct effect on long-term student outcomes.

FOYC is an evidence-based, life skills curriculum designed to reduce risk taking behaviors related to HIV/STI transmission and teen pregnancy. Woven throughout FOYC is a decision making model that provides guidance and practice in problem solving with a focus on how to obtain factual information on sexual health. FOYC was adapted from Focus on Youth (FOY). FOY and Informed Parents and Children Together (CImPACT) were selected to be part of the Centers for Disease Control and Prevention’s “Diffusion of Effective Behavioral Interventions (DEBI)” Portfolio. Detailed information about intervention activities are described elsewhere [22, 23]. Longitudinal evaluations showed that the intervention significantly increased Bahamian youth’s HIV/AIDS knowledge, perceptions of their ability to use condoms, and condom-use intention [24] with evidence of increased condom use [25].

Beginning in 2011, the FOYC curriculum was integrated into the national Health and Family Life Education (HFLE) curriculum and taught by grade 6 teachers among all grade 6 youth in all government primary schools in The Bahamas throughout the school year. The FOYC curriculum consists of eight sessions (each averaging 45 min to 1.5 h in length). Subsequently as the youth reached grade 7 and then grade 8, they received an annual 1-h booster session reviewing decision-making tools, condom use skills and communication skills. Per standard Ministry of Education (MOE) procedure, the grade 6, grade 7, and grade 8 teachers had all been invited to participate in teacher training workshops; for the grade 6 teachers, the training lasted for 1 to 4 days, while for the grade 7 and grade 8 teachers, the training was 1 day. Details of the teachers training are described in our recent publications [23, 26].

National implementation of FOYC in The Bahamas offers a unique opportunity to examine teachers’ pattern of implementation over time and long-term program outcome, which has not been addressed in our prior analyses [23, 26]. Drawing on data gathered through four waves of national implementation, this analysis addresses three research questions: (1) To what extent did the teachers implement the FOYC-booster program after initial implementation of FOYC in grade 6?; (2) What factors influenced the teachers’ patterns of implementation and what is the relationship between influencing factors?; and, (3) How does teachers’ initial implementation of FOYC and subsequent delivery of the booster sessions impact students’ outcomes in grades 6, 7, and 8?

## Methods

### Study site

Beginning in 2011, 80 government elementary schools and 34 government middle (junior high) schools in the Commonwealth of The Bahamas participated in national implementation of FOYC. The 80 schools are located on 14 of the major islands constituting The Bahamas, where more than 98% of the population resides. The 80 participating elementary schools housed 208 grade 6 teachers; the 34 middle schools housed 75 grade 7 teachers and 58 grade 8 teachers.

### Recruitment procedure

Our local research team met several times with the director of education of the Ministry of Education (MOE), district superintendents, and school administrators to discuss this school-based research project at the beginning of this study. Administrators from 117 government primary/junior high schools were contacted and 114 provided written approval of inclusion of their schools (97.4%). Our research team partnered with the Health and Family Life Education (HFLE) unit of MOE to conduct FOYC training workshops for teachers. All HFLE teachers were invited to participate in the training and received information about the research. Two hundred and sixty primary/junior high school teachers were contacted; 251 (96.5%) provided written consent to participate.

### Measures

#### Implementation dose and fidelity of implementation

Grade 6 teachers were asked to complete a teacher implementation checklist specific for each of the eight sessions of FOYC after they had taught the session. The checklist includes all 46 activities in the FOYC curriculum, 30 of which were identified by the developers as “core activities” [i.e., those activities believed to be critical to the effectiveness of the intervention [27]. The teachers indicated which activities they had and had not taught in each session. Implementation dose was defined as the number of core activities (from among a total of 30) actually taught. For those core activities that they taught, the teachers recorded whether they had modified the format of the activity as outlined in the manual to determine *fidelity of implementation.* Student engagement (few, some, and most) and level of comfort (not comfortable, rather comfortable, and very comfortable) in teaching each activity were also recorded. Grade 7 and grade 8 teachers were asked to complete teacher implementation checklist specific for each of the five core activities in the FOYC booster session. The teachers indicated which activities they had and had not taught; for those core activities that they taught, the teachers recorded whether they had modified the format of the activity, student engagement, and their comfort level in teaching the activity.

### Factors associated with implementation

At pre- and post-intervention delivery, all grade six participating teachers were asked to complete questionnaires assessing factors described in the prior research as influencing fidelity of intervention implementation. Information was collected on teacher’s level of formal education (associate degree, teaching certificate, bachelor degree, master degree, doctoral degree), years as a teacher/guidance counselor (1–2 years, 3–5 years, 5–10 years, 10–20 years, >20 years), teacher’s attendance at FOYC training workshop (1 = did not attend, 2 = attended part of a training workshop, 3 = fully attended a training workshop), training in interactive methods (1 = none, 2 = a little, 3 = some, 4 = a lot), prior experience of teaching FOYC or other HIV prevention programs (yes/no), teachers’ attitude towards FOYC/HIV prevention intervention measured as their perceptions of the importance of prevention programs, HIV prevention and the FOYC intervention (1 = not important, 2 = somewhat important, 3 = very important), comfort level with the FOYC curriculum (e.g., “how comfortable do you think you will feel in teaching the materials in FOYC?” 1 = not at all, 2 = somewhat comfortable, 3 = very comfortable), and teacher’s sense of “ownership” of the curriculum [e.g., a belief that the intervention addresses a local issue and reflects Bahamian values and input [7, 12]. The Cronbach’s alpha for perceptions of program importance (five items) was 0.75.

### Teachers’ patterns of initial implementation (high/moderate/low implementers)

As reported previously [23], cluster analysis was used to identify teachers’ patterns of implementation based on implementation dose (i.e., number of core activities taught) and fidelity of implementation (i.e., percentage of core activities being changed during the implementation). Results of the cluster analysis indicated three distinct implementation clusters of teachers: (1) high implementation group (63 teachers, 32%), characterized by high levels of sustained implementation and fidelity of implementation. Teachers in this group taught over 80% of core activities (25 out of 30 core activities) on average and changed only 14% of the core activities; (2) moderate implementation group (105 teacher, 53%), showing moderate levels of sustained implementation but high levels of fidelity of implementation. On average, teachers in this group taught less than half of the core activities (12.3 out of 30 core activities) and changed 13% of the core activities; and 3) low implementation group (31 teachers, 16%), demonstrating low levels of sustained implementation and fidelity. On average, these teachers taught less than one-third of core activities (8.6 out of 30 core activities) and changed three-quarters of the core activities that they taught in the classroom.

### Student outcomes

An anonymous curricular assessment instrument with identifying information only at the level of the school and classroom/teacher, adapted by the MOE from a version of the Bahamian Youth Health Risk Behavioral Inventory (BYHRBI) [22] was administered to grade 6, grade 7, and grade 8 students. The instrument includes a scale of 15 true/false statements to assess level of HIV/AIDS knowledge (“knowledge”) (Cronbach’s *α* = 0.85); a six-item adaptation of the Condom-use Skills Checklist (Cronbach’s *α* = 0.83) [28] to assess condom-use knowledge and skills (“reproductive health skills”); a three-item self-efficacy scale regarding pregnancy/STI prevention methods (“self-efficacy”) (Cronbach’s *α* = 0.81); and, one question assessing the youth’s likelihood of using a condom if he/she were to engage in sexual intercourse within the next 6 months (“intention to use protection”) [five-point Likert scale ranging from 1 (very unlikely) through 5 (very likely)].

#### Analysis

Descriptive statistics (mean and standard deviation) of HIV/AIDS knowledge, condom-use skills, self-efficacy, and intention to use protection were calculated for whole sample and stratified by teachers’ patterns of initial implementation (high/moderate/low implementers) at baseline, and grade 6, grade 7, and grade 8 follow-ups. A line chart was then constructed to graphically display the longitudinal trends of mean scores of these student outcomes. The difference in student outcomes across the four time points were examined using ANOVA with Tukey honestly significant difference (HSD) tests, using the whole sample and stratified by the implementation group. The difference in knowledge, skills, self-efficacy, and intention across the three implementation groups at each time point was assessed using ANOVA. To examine the associations of grade 7 and grade 8 teacher delivery of booster sessions with grade 8 student outcomes, students’ knowledge, skills, self-efficacy, and intentions were compared according to their grade 7 and grade 8 teachers’ levels of implementation of the booster sessions (categorized into three groups: taught 0–1 activity, 2–3 activities or 4–5 activities) using ANOVA with the Tukey HSD post hoc tests.

Pearson (for continuous variables) and Spearman (for ordinal variables) correlation analyses were conducted to examine the associations between factors influencing teacher’s patterns of implementation and student outcomes. The anonymous student questionnaires were not linked at the level of the individual student; however, the questionnaires were linked to the teacher (classroom). Thus, we calculated average scores of student outcomes for each teacher in correlation analyses.

We further examined the association of grade 6 teachers’ patterns of implementation (high/moderate/low implementers) with grade 8 student outcomes using mixed-effects modeling, adjusting for clustering effects of classroom (teacher) and/or school. Independent variables included teacher’s implementation clusters, student’s age, sex, and baseline student outcomes. School and class were included as random effect variables in the model. Similarly, the effect of grade 7 and grade 8 teacher delivery of booster sessions on student outcomes was further assessed using mixed effects models, controlling for clustering effects of classroom and/or school and baseline difference.

To examine the effects of both the grade 6 teachers’ implementation of the FOYC intervention and subsequent delivery of the booster session in grade 7 and grade 8 on long-term student outcomes in grade 8, we expanded the mixed-effects models described above by including both grade 6 teachers’ patterns of implementation and grade 7 and grade 8 teacher’s level of implementation of booster session in the same model. We further examined the relationship of different combinations/scenarios of grade 6 implementation of FOYC (high/moderate/low implementers) and grade 7 and grade 8 teacher’s delivery of booster session [poor (0–3 activities)/fair (4–7 activities)/good (8–10 activities) delivery] with student outcomes using mixed-effects models controlling for clustering effects and baseline difference. All analyses were performed using SAS 9.4 statistical software package (SAS Institute Inc., Cary, NC, USA).

Structural equation modeling (SEM) analysis was conducted to examine the relationships among factors influencing teacher’s patterns of implementation, and student outcomes using the Mplus 7 with multilevel add-on. A starting model (implementation model) was estimated to examine the interrelationships among factors influencing teacher’s patterns of implementation and their direct and indirect effects on implementation patterns. Subsequently, a full model was constructed by including grade 6, grade 7, and grade 8 student outcome latent variables, and implementation of grade 7 and grade 8 booster sessions into the revised implementation model. Since students were clustered within classes in 80 schools, the cluster option in Mplus was used to correct for the potential underestimation of standard errors [29]. Standardized regression coefficients for all paths were estimated using robust maximum likelihood (MLR) estimation. Missing data was handled using full information maximum likelihood (FIML). Goodness of model fit was assessed using chi-square to degrees-of-freedom ratio ($\chi$ ^2^/df), root mean square error of approximation (RMSEA), Bentler’s comparative fit index (CFI) and Tucker Lewis Index (TLI) [30]. Standardized path coefficients were presented in Fig. 3.

## Results

### Study participants

Data were collected from 208 grade 6 teachers, 75 grade 7 teachers, 58 grade 8 teachers and 4411 grade 6 students at baseline; from 4,168 (94.5%) of all grade 6 students at the grade 6 follow-up; from 3,439 (78.0%) students at the grade 7 follow-up; and, from 3256 (73.8%) students at the grade 8 follow-up. The average age of the students at baseline was 10.4 (SD = 1.7) years. The vast majority (>95%) of students are of African descent and about one-half are female.

### Teachers’ implementation of the booster sessions after initial implementation of FOYC

Among the 75 grade 7 teachers who participated in the national implementation study, 18 teachers taught 4 or 5 activities (from among 5 activities) in the booster session, 29 taught 2 or 3 activities and 28 taught none or only one activity. Among the 58 grade 8 teachers who were involved in the current study, 27 teachers taught 4 or 5 activities in the booster session, 25 taught 2 or 3 activities and 6 taught none or only one activity.

### Changes in mean scores for HIV/AIDS knowledge, reproductive health skills, self-efficacy, and intention to use protection by three implementation groups

**Fig. 1:**
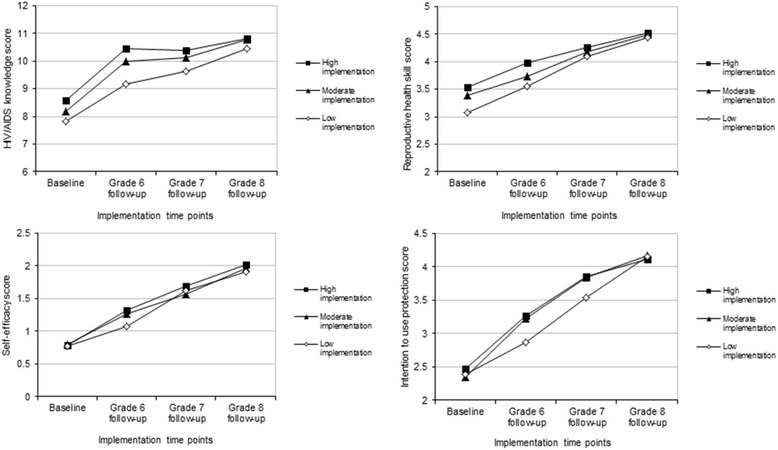
Change in HIV/AIDS knowledge, reproductive health skills, self-efficacy, and intention to use protection from baseline to 24-month follow-up, stratified by grade 6 teacher implementation cluster

Figure 1 displays changes in the mean scores of HIV/AIDS knowledge, reproductive health skills, self-efficacy and intention to use protection over time according to the implementation-level of the grade 6 teacher who were categorized into three implementation groups: high or moderate or low implementation. Overall, knowledge, skills, self-efficacy and intention increased significantly across the four time points for all three implementation groups except that the increases in knowledge from grade 6 follow-up to grade 7 follow-up were not significantly different in the high and moderate implementation groups. A large increase in knowledge in the high and moderate implementation groups occurred at the grade 6 follow-up; this increase resulted in an upward displacement of the curves for the two implementation groups. After the grade 6 follow-up, the trajectories were relatively flat for the high and moderate implementation groups, although their upward displacement was retained throughout the follow-up period. Reproductive health skills were higher among students who had been taught by grade 6 high and moderate implementation teachers than those with grade 6 low implementation teachers at baseline and the grade 6 and grade 7 follow-ups. At the grade 8 follow-up, the mean difference in skills became statistically not significant. Comparable at baseline, self-efficacy and intention were higher among students with grade 6 high and moderate implementation teachers than those with grade 6 low implementation teachers at the grade 6 and/or grade 7 follow-ups; at grade 8 follow-up there were no significant differences in self-efficacy and/or intentions among the three implementation groups.

### Effect of a brief FOYC-booster program on grade 8 student outcome according to grade 6 teacher FOYC implementation patterns

Students’ HIV/AIDS knowledge, reproductive health skills, self-efficacy and intention to use protection were significantly associated with grade 8 teachers’ level of implementation of the booster session (Table 1). Overall, knowledge, skills, self-efficacy, and intention were the highest among youth whose teachers covered four to five activities (“good” implementation) of booster session, followed by youth whose teachers covered two to three activities (“fair” implementation), with youth whose teachers did not teach or only taught one activity (“poor” implementation) of the booster session demonstrating the lowest scores. In stratified analysis among youth whose grade 6 teachers were in the *low* implementation group, those whose grade 8 teachers exhibited good and/or fair implementation of the booster compared to those whose grade 8 teachers exhibited poor implementation demonstrated greater increases in three of the four student outcomes in grade 8 (knowledge: 10.8 vs. 10.3 vs. 9.6, *F* = 5.41, *P* < 0.01; skills: 4.6 vs. 4.3 vs. 4.2, *F* = 3.82, *P* = 0.023; self-efficacy: 2.0 vs. 1.9 vs. 1.4, *F* = 4.88, *P* < 0.01). Among youth whose grade 6 teachers were in the *moderate* implementation group, those whose grade 8 teachers exhibited good and/or fair booster implementation compared to those whose grade 8 teachers exhibited poor implementation demonstrated greater increase in reproductive health skills and intention (skills: 4.6 vs. 4.4 vs. 4.4, *F* = 4.67, *p* < 0.05; intention: 4.3 vs. 4.2 vs. 3.6, *F* = 13.41, *P* < 0.001). Among youth whose grade 6 teachers were in the *high* implementation group, those whose grade 8 teachers exhibited poor and/or fair booster implementation compared to those grade 8 teachers who exhibited good implementation demonstrated poorer reproductive health skills (skills: 4.4 vs. 4.4 vs. 4.6, *F* = 4.88, *P* < 0.01).

**Table 1:** Associations grade 8 teachers’ implementation of booster sessions with student outcomes

Variables	Number of activities in the booster session completed			*F*	Post-hoc comparison^b^
	0~1 activities (1)	2~3 activities (2)	4~5 activities (3)		
Sample size (*n*)^a^	296	1104	1705		
HIV/AIDS knowledge (range 0~15 points)	10.45(2.39)	10.65(2.19)	10.85(2.12)	5.72^**^	(1,3) (2,3)
High implementation group	10.85(1.99)	10.70(2.21)	10.87(2.08)	0.71	
Moderate implementation group	10.46(2.54)	10.73(2.09)	10.87(2.14)	2.37	
Low implementation group	9.55(2.40)	10.34(2.49)	10.78(2.04)	5.41^**^	(1,3)
Preventive reproductive health skills (range 0~6 points)	4.34(1.13)	4.37(1.15)	4.59(1.07)	16.95^***^	(1,3) (2,3)
High implementation group	4.37(1.11)	4.42(1.15)	4.62(1.06)	4.88^**^	(2,3)
Moderate implementation group	4.39(1.11)	4.39(1.14)	4.56(1.09)	4.67^*^	(2,3)
Low implementation group	4.22(1.24)	4.34(1.22)	4.57(1.07)	3.82^*^	(1,3) (2,3)
Self-efficacy (range 0~3 points)	1.82(1.12)	1.95(1.12)	2.05(1.08)	6.43^***^	(1,3)
High implementation group	1.94(1.06)	1.95(1.13)	2.09(1.06)	2.16	
Moderate implementation group	1.85(1.10)	1.96(1.09)	2.01(1.09)	1.44	
Low implementation group	1.39(1.30)	1.94(1.18)	2.01(1.11)	4.88^**^	(1,2) (1,3)
Intention to use protection (range 1~5 points)	3.81(1.63)	4.21(1.46)	4.17(1.47)	8.58^**^	(1,2) (1,3)
High implementation group	4.12(1.49)	4.21(1.47)	4.07(1.55)	0.90	
Moderate implementation group	3.56(1.74)	4.18(1.47)	4.26(1.40)	13.41^***^	(1,2) (1,3)
Low implementation group	3.97(1.46)	4.26(1.45)	4.10(1.52)	0.80	

*P < 0.05

**P < 0.01

***P < 0.001

^a^151 youth who did not identify their grade 8 teachers were excluded

^b^Numbers in parentheses indicating significantly different groups based on post-hoc analysis

The results of the mixed-effects models indicate that the quality of grade 6 teachers’ implementation was significantly related to increased HIV/AIDS knowledge and self-efficacy among grade 8 students (knowledge: *β* = 0.37, SE = 0.19, *p* = 0.05; self-efficacy: *β* = 0.18, SE = 0.07, *p* < 0.01). Changes in students’ reproductive health skills and intention to use protection were not related to grade 6 teacher implementation. In summary, youth whose teachers belonged to the high implementation group demonstrated better student outcomes in grade 8 than those youth whose teachers were in the low implementation group (Additional file 1). Similarly, grade 7 teachers’ level of implementation of the booster session was significantly related to grade 8 student improvements in HIV/AIDS knowledge and self-efficacy (Additional file 2). The results of the mixed-effects models show that grade 8 teachers’ level of implementation of the booster session was significantly related to grade 8 student improvements in reproductive health skills, condom use self-efficacy, and intention to use protections (skills: β = 0.24, SE = 0.08, p,0.01; self-efficacy: *β* = 0.22, SE = 0.07, *p* < 0.01; intention: β = 0.35, SE = 0.11, p,0.001); the relationship of the students’ HIV/AIDS knowledge with grade 8 teacher implementation of the booster sessions was marginally significant (Additional file 3).

### Effects of grade 6 teacher’s initial implementation of FOYC intervention and grade 7 and 8 teacher’s delivery of booster session on long-term student outcomes

Table 2 presents the relationship of different combinations of grade 6 implementation of FOYC and grade 7 and grade 8 delivery of the booster sessions with student outcomes. Compared to youth whose grade 6 teachers belonged to the low implementation group and whose grade 7 and grade 8 teachers exhibited poor booster implementation (“poor-performing teachers”), youth whose grade 6 teachers belonged to the high or moderate implementation groups and whose grade 7 and grade 8 teachers exhibited good or fair booster implementation performed better in three of the four student outcomes (including HIV/AIDS knowledge, reproduction health skills and self-efficacy). Presence of poor-performing teachers in either grade 6 or in grade 7 and/or 8 were mitigated by better performing teachers in the alternate year(s). Thus, compared to youth with poor-performing teachers in grades 6, 7 and 8, youth whose grade 6 teachers belonged to the high or moderate implementation groups *but* whose grade 7 and grade 8 teachers exhibited poor booster implementation performed better in HIV/AIDS knowledge only; youth whose grade 6 teachers were in the low implementation group but whose grade 7 and grade 8 teachers exhibited good or fair booster implementation performed better in HIV/AIDS knowledge only. Youth whose grade 6 teachers belonged to the high or moderate implementation groups and whose grade 7 and grade 8 teachers exhibited good or fair booster implementation demonstrated better long-term student outcomes than youth whose grade 6 teachers were in the low implementation group and whose grade 7 and grade 8 teachers exhibited poor booster implementation.

**Table 2:** Mixed-effects models assessing the effects of *different combinations* of grade 6 teacher’s implementation of FOYC intervention and grade 7 and 8 teacher’s delivery of booster session on student outcomes

Variables	Estimated models											
	HIV/AIDS knowledge			Preventive reproductive health skills			Self-efficacy			Intention to use protection		
	*B*	SE	*t*	β	SE	*t*	*β*	SE	*t*	β	SE	*t*
*Fixed effect*												
Intercept	9.565	0.384	24.91^***^	4.237	0.189	22.43^***^	1.818	0.173	10.51^***^	4.417	0.259	17.04^***^
Age	0.014	0.024	0.59	−0.011	0.013	−0.84	−0.015	0.013	−1.22	−0.057	0.017	−3.34^**^
Gender												
Male	0.014	0.079	0.17	0.018	0.042	0.44	0.029	0.42	0.70	0.083	0.058	1.44
Female (ref)												
Baseline student outcome	0.027	0.016	1.70^#^	0.029	0.016	1.76^#^	0.017	0.020	0.84	0.036	0.017	2.10^*^
Nine combinations of grade 6 implementation of FOYC and grade 7 and grade 8 booster delivery												
High implementation of FOYC and good delivery of booster in grade 7/8	0.984	0.325	3.03^**^	0.380	0.155	2.46^*^	0.363	0.134	2.70^**^	0.002	0.228	0.01
High implementation of FOYC and fair delivery of booster in grade 7/8	0.841	0.294	2.86^**^	0.319	0.139	2.30^*^	0.362	0.122	2.98^**^	0.186	0.207	0.90
High implementation of FOYC and poor delivery of booster in grade 7/8	0.714	0.342	2.09^*^	0.132	0.164	0.80	0.213	0.146	1.46	0.228	0.241	0.95
Moderate implementation of FOYC and good delivery of booster in grade 7/8	0.772	0.317	2.44^*^	0.416	0.153	2.71^**^	0.311	0.137	2.27^*^	0.405	0.225	1.80^#^
Moderate implementation of FOYC and fair delivery of booster in grade 7/8	0.856	0.284	3.02^**^	0.278	0.135	2.06^*^	0.318	0.119	2.67^**^	0.251	0.201	1.25
Moderate implementation of FOYC and poor delivery of booster in grade 7/8	0.767	0.319	2.40^*^	0.279	0.155	1.80^#^	0.138	0.138	1.00	−0.035	0.227	−0.15
Low implementation of FOYC and good delivery of booster in grade 7/8	1.057	0.509	2.08^*^	0.357	0.256	1.40	0.370	0.253	1.46	0.580	0.364	1.59
Low implementation of FOYC and fair delivery of booster in grade 7/8	0.590	0.282	2.09^*^	0.190	0.143	1.32	0.248	0.131	1.89^#^	0.181	0.207	0.88
Low implementation of FOYC and poor delivery of booster in grade 7/8 (ref)												
*Random effect*												
School^a^	0.026	0.049	0.52	0.015	0.009	1.55^#^	-	-		0.009	0.020	0.43
Class (nested within school)^a^	0.384	0.083	4.65^***^	0.015	0.012	1.27	0.005	0.009	0.61	0.142	0.035	4.00^***^

^#^ *P* < 0.10

*P < 0.05

**P < 0.01

***P < 0.001

^a^ *z* test. Good delivery of booster = covered 8–10 activities; fair delivery of booster = covered 4–7 activities; poor delivery of booster = covered 0–3 activities

### Bivariate correlation among factors influencing implementation and student outcomes

Grade 6 teacher’s level of comfort with the FOYC curriculum, attitudes towards the intervention, attendance of FOYC training workshop were positively associated with teacher implementation cluster (*r* = 0.24~0.34, *p* < 0.01). Teacher’s level of comfort with FOYC was positively associated with attitudes towards FOYC, training in interactive teaching and years as teacher (*r* = 0.20~0.34, *p* < 0.01). In addition, training in interactive teaching was positively associated with attendance of FOYC workshop and implementation cluster (*r* = 0.27~0.30, *p* < 0.001). Students’ HIV/AIDS knowledge, reproductive health skills, self-efficacy and intention to use protection were significantly correlated with each other in grade 6 (*r* = 0.16~0.44, *p* < 0.05) and grade 7 (*r* = 0.15~0.49, *p* < 0.05). Grade 8 students’ HIV/AIDS knowledge, reproductive health skills and intention to use protection (except self-efficacy) were significantly correlated (*r* = 0.22~0.41, *p* < 0.01). Grade 6 students’ knowledge, skills, self-efficacy and intention were significantly correlated with grade 7 and grade 8 students’ knowledge, skills, self-efficacy and intention, respectively (*r* = 0.22~0.61, *p* < 0.01). Grade 7 teacher’s delivery of the booster session was significantly correlated with grade 7 students’ knowledge and skills and grade 8 students’ knowledge (*r* = 0.19~0.23, *p* < 0.01). Grade 8 teacher’s delivery of the booster session was significantly correlated with grade 8 students’ knowledge and skills (*r* = 0.14, *p* < 0.05). Grade 7 teacher’s level of implementation of booster session is positively associated with grade 8 teacher’s level implementation of booster session (Table 3).

**Table 3:** Correlation coefficients among factors influencing teacher’s implementation of FOYC and student outcomes

Variables	1	2	3	4	5	6	7	8	9	10	11	12	13	14	15	16	17	18	19	20	21
Factors influencing implementation																					
1. Comfort level with FOYC	1																				
2. Attitudes towards FOYC	0.20^a^	1																			
3. Student engagement	0.07	0.20^b^	1																		
4. Interactive teaching	0.34^c^	0.18^a^	0.01	1																	
5. Years as teacher	0.33^c^	0.02	0.08	0.09	1																
6. FOYC workshop	0.13	0.13	0.09	0.30^c^	0.04	1															
7. Implementation cluster	0.24^b^	0.35^c^	0.14^a^	0.27^c^	-0.08	0.25^c^	1														
Student outcomes in grade 6																					
8. HIV/AIDS knowledge	0.07	0.32^c^	0.02	0.03	-0.02	0.12	0.36^c^	1													
9. Reproductive health skills	0.11	0.30^c^	0.09	0.12	0.10	0.11	0.35^c^	0.44^c^	1												
10. Self-efficacy	0.11	0.27^c^	0.04	0.11	0.10	0.09	0.16^a^	0.19^a^	0.35^c^	1											
11. Intention	0.15	0.24^b^	0.10	0.12	0.02	0.05	0.18^a^	0.39^c^	0.22^b^	0.16^a^	1										
Student outcomes in grade 7																					
12. HIV/AIDS knowledge	0.13	0.30^c^	0.07	0.02	0.14	0.16^a^	0.20^b^	0.47^c^	0.28^c^	0.10	0.26^c^	1									
13. Reproductive health skills	0.05	0.04	0.12	0.06	0.11	0.10	0.05	0.22^b^	0.22^b^	0.01	0.18^a^	0.48^c^	1								
14. Self-efficacy	0.02	-0.00	0.11	0.00	0.03	-0.07	-0.01	0.09	0.23^b^	0.48^c^	0.08^c^	0.15^a^	0.15^a^	1							
15. Intention	0.08	0.12	0.18^a^	0.05	0.00	-0.03	0.08	0.32^c^	0.20^b^	0.05	0.41^c^	0.41^c^	0.49^c^	0.30^c^	1						
Student outcomes in grade 8																					
16. HIV/AIDS knowledge	0.04	0.19^a^	0.07	-0.03	0.17^a^	0.02	0.10	0.40^c^	0.27^c^	-0.06	0.36^c^	0.61^c^	0.33^c^	-0.05	0.33^c^	1					
17.Reproductive health skills	-0.07	0.12	-0.03	0.11	0.18^a^	0.07	0.13	0.06	0.23^b^	-0.03	0.21^b^	0.16^a^	0.32^c^	-0.06	0.15^a^	0.38^c^	1				
18. Self-efficacy	0.04	0.11	0.05	0.11	0.22^b^	0.04	0.09	0.09	0.26^c^	0.32^c^	0.05	0.10	0.22^b^	0.48^c^	0.27^c^	0.03	0.41^c^	1			
19. Intention	-0.03	0.11	0.09	0.06	-0.10	-0.01	-0.07	0.24^b^	0.11	-0.17^a^	0.45^c^	0.31^c^	0.16^a^	-0.03	0.39^c^	0.36^c^	0.22^b^	-0.04	1		
Delivery of booster session																					
20. Grade 7 booster	0.06	0.12	0.03	0.07	0.09	0.05	0.12	0.17^a^	0.23^b^	0.19^a^	0.16^a^	0.20^c^	0.23^c^	0.05	0.10	0.19^c^	0.13	0.09	0.08	1	
21. Grade 8 booster	0.02	-0.01	0.07	-0.02	0.09	-0.05	0.07	0.09	0.12	0.07	0.09	0.16^a^	0.04	0.07	0.09	0.14^a^	0.14^c^	0.06	0.06	0.15^a^	1

^a^ *P* < 0.05

^b^ *P* < 0.01

^c^ *P* < 0.001

### Relationships among factors influencing teacher’s patterns of implementation and the effects of initial implementation and subsequent booster sessions on student outcomes

**Fig. 2:**
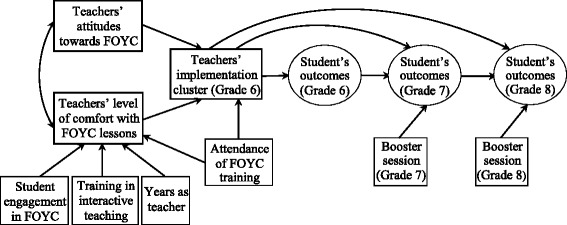
Hypothesized structural model of relationships among factors that influence teachers’ quality of implementation and long-term student outcomes

An initial hypothetical model was developed based on a synthesis of the empirical literature (Fig. 2). The model posits that teachers’ attitudes towards the intervention (i.e., perception of the importance of FOYC) and their levels of comfort with delivering the FOYC lessons have a direct positive effect on implementation patterns (high/moderate/low implementers), which in turn affect student outcomes. Teacher’s level of comfort is influenced by whether they received training in the delivery of the FOYC curriculum and/or interactive teaching, perceived student engagement in FOYC, and their duration as a teacher. Teachers’ attendance at the FOYC training workshop is also hypothesized to have a direct effect on their implementation. Estimation of this model revealed a significant chi-square statistic and unacceptable CFI and TLI values (close to 0.80). In modifying the initial model, we eliminated two nonsignificant paths including the paths from student engagement in FOYC and attendance at FOYC training to teachers’ level of comfort with FOYC. The revised model was then estimated and revealed acceptable model fit. This revised implementation model was expanded into a full model by including student outcomes and grade 7 and grade 8 booster session. In modifying the full model, we eliminated one nonsignificant path from teachers’ implementation quality in grade 6 to grade 8 student outcome and added a path from teachers’ attitudes towards FOYC (perception of the importance of FOCY) to grade 6 student outcome. Self-efficacy as one of the four indicators for the student outcome latent variable was removed due to low factor loadings (ranging from 0.28 to 0.38). The overall fit of the revised model was good. The chi-square/df ratio was 1.67, the RMSEA was 0.06, the CFI was 0.93, and the TLI was 0.91.

**Fig. 3:**
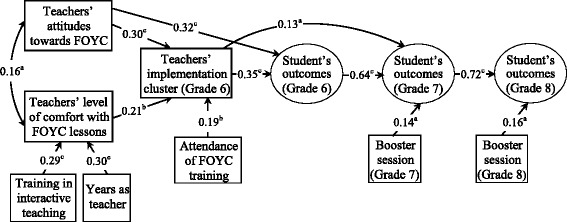
Revised structural model showing relationships among factors that influence fidelity of implementation and long-term student outcomes (including HIV/AIDS knowledge, condom use skills and intention to use protection). (Model fit: CFI = 0.930; TLI = 0.907; RMSEA = 0.058; Chi-Square/DF = 1.67). *R* ^2^ value for grade 6, grade 7, and grade 8 student outcomes is 0.30, 0.41, and 0.52, respectively

The revised structural model demonstrated relationships among factors and their direct and indirect effect on implementation patterns and student outcomes (Fig. 3). There were four manifest exogenous variables, two manifest endogenous (i.e., comfort level and implementation cluster) and three latent endogenous variables (e.g., student’s outcomes in grade 6, 7 and 8) in the model. In the final model, teachers’ attitudes towards the intervention, comfort level with the FOYC curriculum and attendance of FOYC training predicted high quality of implementation, which in turn predicted better student outcomes (HIV/AIDS knowledge, reproductive health skills, and intention to use protection). More years as a teacher or guidance counselor and training in interactive teaching were positively associated with teachers’ level of comfort with FOYC. In addition, teacher’s perception of the importance of the FOYC intervention had a direct positive effect on grade 6 student outcomes. Grade 6 student outcome, teachers’ implementation cluster and grade 7 teachers’ delivery of booster session predicted grade 7 student outcome. Grade 7 student outcome and grade 8 teachers’ delivery of booster session predicted grade 8 student outcome. The analysis revealed an *R* ^2^ value of 0.20 for implementation cluster and of 0.30, 0.41, and 0.52 for grade 6, grade 7, and grade 8 student outcomes, respectively.

The Sobel test of mediation effect indicated that implementation cluster mediated the relationship between teacher’s level of comfort with the FOYC curriculum and student’s outcome (*z* = 2.51, *p* < 0.05). In addition, the Sobel test suggested that grade 6 implementation quality partially mediates the relationships between teachers’ attitudes towards the intervention and student outcome (*z* = 3.21, *p* < 0.01).

## Discussion

The current study examined the interrelationships among multiple program provider-level factors and how these processes influence teachers’ patterns of implementation (measured by high/moderate/low implementation clusters) of an evidence-based sexual risk reduction program in 80 government elementary schools and 34 government middle schools in The Bahamas across 30 months of follow-up. We also assessed the impact of initial implementation and subsequent delivery of the booster sessions on long-term program outcomes. We found that several provider-level factors were associated with implementation patterns, and high-quality initial implementation was significantly related to short-term program outcomes. Level of implementation of the booster sessions was significantly related to subsequent student outcomes. Both initial implementation and subsequent delivery of booster sessions are of critical importance in achieving long-term program outcomes.

Several teacher-level factors were found to be significantly related to teachers’ initial implementation of FOYC. Consistent with previous research [10, 12, 16], we found that teachers’ attitudes towards the prevention program, their comfort level with the intervention curriculum, and teachers’ attendance at curriculum training workshop are strong predictors of the teacher’s quality of implementation (defined by high degrees of implementation and fidelity of implementation). Higher levels of comfort among teachers with the curriculum may lead to greater confidence in their ability to conduct the intervention sessions (including sensitive topics such as condom use, teen pregnancy and sexual harassment/abuse) in the classroom. Teachers who hold favorable attitudes towards the intervention perceive the importance of HIV prevention in schools, and thus they are more likely to implement more sessions without significantly modifying the format of the activity. Teachers who attended curriculum training workshops were more familiar with the contents of the intervention curriculum and acquired interactive teaching skills. Further, teachers’ attendance at training workshops may reflect positive attitudes towards the prevention program. Teachers’ comfort with the intervention curriculum also demonstrated a significant mediating effect on the relationship between training in interactive teaching methods and implementation fidelity. Inconsistent with previous studies [16], our study did not find significant association between student engagement in the intervention curriculum and teachers’ quality of implementation. This could be caused by small variation in teachers’ reports of student engagement (as the vast majority of teachers said “most” students were engaged in the lessons). Teachers’ attitudes towards a prevention program, self-efficacy in teaching intervention curriculum and skills in interactive teaching are potential modifiable factors related to program delivery [7]. Therefore, pre-implementation teacher training should place greater emphasis on enhancing teachers’ competency in teaching the intervention curriculum, shaping teachers’ belief about the importance of HIV prevention, and demonstrating and reinforcing the use of interactive methods in the context of prevention programs.

Both teachers’ initial implementation of FOYC and subsequent delivery of the booster sessions are related to students’ outcomes. As previously reported [23, 26], high quality of initial implementation (characterized by high levels of implementation and fidelity of implementation) was significantly related to all four student outcomes 6 months after intervention implementation and related to two student outcomes (HIV/AIDS knowledge and intention to use protection) 18 months after initial implementation. Data in the present study indicate that the quality of initial implementation had little impact on long-term student outcomes (e.g., 30 months after implementation, it was only significantly related to student self-efficacy). The level of implementation of the booster session in grade 8 was significantly related to three student outcomes (reproductive health skills, self-efficacy and intention to use protection). Simultaneous examination in the mixed-effects model of the effects of grade 6 initial implementation of FOYC and grade 7 and grade 8 delivery of the booster sessions on student outcomes revealed that high quality of initial implementation combined with good delivery of the booster sessions resulted in improvements in three outcomes (knowledge, skills and self-efficacy) whereas high quality of initial implementation combined with poor delivery of the booster sessions only resulted in improvement in one outcome (knowledge) in comparison to poor quality of initial implementation and poor delivery of booster sessions. These findings suggest that the effect of initial implementation on long-term program outcome is fading without quality delivery of booster program, which highlights the importance of the FOYC-booster program after the intervention implementation.

While longitudinal trends of mean scores for knowledge, skills, self-efficacy, and intention show consistent increases for all three implementation groups, trajectory patterns of HIV/AIDS knowledge show that the increases in knowledge from the grade 6 follow-up to the grade 7 follow-up were not significantly different in the high and moderate implementation groups. This lack of difference may be because the high and moderate implementation groups gained large increase in knowledge during the grade 6 implementation phase, e.g., there was a “ceiling effect” for knowledge for both groups between grade 6 and grade 7 follow-ups.

Several potential limitations should be noted in this study. First, our findings were based on teachers’ and students’ self-reports, which are subject to social desirability and recall bias. It is possible that teachers over-reported their level of implementation of the intervention curriculum and booster sessions and provided responses that they thought would be more appropriate (as teachers were expected to teach the intervention curriculum as integrated components of Health and Family Life Education class). In the current study, trained observers independently observed and assessed approximately 20% of each teacher’s classes and booster sessions. We compared the teacher and observer reports on activities taught in these sessions and found that the teacher-observer agreement was high (over 80%), indicating that teacher’s self-reports are reliable [31]. Second, typical of many studies of this kind, there was relatively high attrition rate (i.e., about 20%) to grade 7 follow-up. The major reasons for attrition in grade 7 included students’ non-identification of their grade 6 teachers in the grade 7 follow-up survey and loss of contact due to students’ graduation from primary school and transferring into non-government middle schools (private or religious-based schools) [26]. Third, most (75%) middle school teachers did not implement all the activities in the booster session, which might compromise long-term effect of the intervention. Incomplete implementation of the booster session may reflect several factors. For example, middle school teachers may be reluctant to reduce time from academic subjects that are assessed through national exams; currently HIV prevention is not included in the middle school national exams. As well, the middle school training for FOYC is substantially shorter than is the training for elementary school teachers. In the absence of equally intensive training, the teachers may not perceive HIV prevention among middle adolescents as being a priority area relative to its importance among elementary school students. Teacher’s training workshops for the middle schools may need to place additional emphasis on the importance of HIV risk reduction among middle adolescents. Fourth, although the literature emphasizes the importance of teachers’ self-efficacy and confidence regarding their ability to implement the program on their actual performance, we did not collect these measures from the teachers. However, we did measure teachers’ comfort level with the FOYC curriculum, which we believe is a close proxy for teacher self-efficacy and confidence. Finally, school-level factors such as support by the principal or school administrators’ perception of importance of HIV prevention were only collected from about half of the participating schools. Thus, these data were not included in the present analysis.

## Conclusions

This study provides an integrated understanding of the relationships between teacher characteristics, training experience and perceptions with quality of implementation, teachers’ subsequent delivery of boost sessions, and long-term student outcomes. This study expands our understanding of the complex ways in which teachers’ attitudes and confidence are influenced by their training and teaching experience, and influence quality of implementation and program outcome. These findings have significant implications for future implementation efforts in school settings. First, given that teachers’ attitudes towards the prevention program and their levels of comfort with the curriculum are the most influential factors on quality of implementation, it is important that teacher pre-implementation training workshop focus on strengthening teachers’ self-efficacy to implement the intervention curriculum with high quality, and enhancing positive beliefs about and promoting positive attitudes towards the prevention program. Second, the brief booster sessions delivered one and two years after the initial implementation demonstrated significant effects on student outcomes, suggesting that booster sessions are important to maintain the long-term program outcome. The quality of booster implementation can significantly impact student outcomes, indicating the critical importance of ongoing teaching training and technical assistance with the booster program after initial implementation.

## Additional files

Additional file 1: Mixed-effects models assessing the effects of *grade 6 teacher’s implementation of FOYC intervention* on long-term student outcomes in grade 8. (DOC 52 kb)
Additional file 2: Mixed-effects models assessing the effects of *grade 7 teacher’s delivery of booster* session on student outcomes in grade 8. (DOC 51 kb)
Additional file 3: Mixed-effects models assessing the effects of grade *8 teacher’s delivery of booster session* on student outcomes in grade 8. (DOC 52 kb)
