# Supplementary material for: Multi-year school-based implementation and student outcomes of an evidence-based risk reduction intervention
Source: Implement Sci. 2017 Feb 10;12:16. doi: 10.1186/s13012-016-0539-7 (PMC5303204; doi:10.1186/s13012-016-0539-7)
Supplement: Additional file 1: — Mixed-effects models assessing the effects of grade 6 teacher’s implementation of FOYC intervention on long-term student outcomes in grade 8. (DOC 52 kb) [file 13012_2016_539_MOESM1_ESM.doc]

| **Additional file 1 Mixed-effects models assessing the effects of *grade 6 teacher’s implementation of FOYC intervention* on long-term student outcomes in grade 8** | | | | | | | | | | | | |
| --- | --- | --- | --- | --- | --- | --- | --- | --- | --- | --- | --- | --- |
| Variables | Estimated models | | | | | | | | | | | |
|  | HIV/AIDS knowledge | | | Preventive reproductive health skills | | | Self-efficacy | | | Intention to use protection | | |
|  | Β | SE | *t* | β | SE | t | β | SE | t | β | SE | *t* |
| *Fixed effect* |  |  |  |  |  |  |  |  |  |  |  |  |
| Intercept | 10.545 | 0.634 | 16.62^***^ | 4.449 | 0.186 | 23.96^***^ | 1.989 | 0.146 | 13.59^***^ | 4.594 | 0.226 | 20.37^***^ |
| Age | -0.032 | 0.055 | -0.59 | -0.012 | 0.013 | -0.96 | -0.014 | 0.013 | -1.09 | -0.056 | 0.018 | -3.19^**^ |
| Gender |  |  |  |  |  |  |  |  |  |  |  |  |
| Male | -0.101 | 0.086 | -1.16 | -0.021 | 0.047 | -0.45 | 0.022 | 0.045 | 0.48 | 0.100 | 0.063 | 1.59 |
| Female (ref) |  |  |  |  |  |  |  |  |  |  |  |  |
| Baseline student outcome | 0.035 | 0.017 | 2.01^*^ | 0.033 | 0.018 | 1.83^#^ | 0.009 | 0.021 | 0.40 | 0.038 | 0.018 | 2.09^*^ |
| Grade 6 teacher’s implementation  clusters |  |  |  |  |  |  |  |  |  |  |  |  |
| High Implementation Group | 0.373 | 0.196 | 1.91^*^ | 0.146 | 0.092 | 1.59 | 0.182 | 0.068 | 2.69^**^ | -0.067 | 0.145 | -0.47 |
| Moderate Implementation Group | 0.336 | 0.187 | 1.79^#^ | 0.109 | 0.088 | 1.23 | 0.124 | 0.066 | 1.89^#^ | 0.052 | 0.139 | 0.37 |
| Low Implementation Group |  |  |  |  |  |  |  |  |  |  |  |  |
| *Random effect* |  |  |  |  |  |  |  |  |  |  |  |  |
| School^†^ | 0.032 | 0.044 | 0.73 | 0.017 | 0.011 | 1.58^#^ | - | - |  | 0.004 | 0.021 | 0.21 |
| Class (nested within school)^†^ | 0.284 | 0.074 | 3.81^***^ | 0.011 | 0.012 | 0.89 | - | - |  | 0.147 | 0.039 | 3.74^***^ |
| # P<0.10; * P<0.05; ** P<0.01; *** P<0.001. ^†^ z test. | | | | | | | | | | | | |
