# Supplementary material for: Multi-year school-based implementation and student outcomes of an evidence-based risk reduction intervention
Source: Implement Sci. 2017 Feb 10;12:16. doi: 10.1186/s13012-016-0539-7 (PMC5303204; doi:10.1186/s13012-016-0539-7)
Supplement: Additional file 2: — Mixed-effects models assessing the effects of grade 7 teacher’s delivery of booster session on student outcomes in grade 8. (DOC 51 kb) [file 13012_2016_539_MOESM2_ESM.doc]

| **Additional file 2 Mixed-effects models assessing the effects of *grade 7 teacher’s delivery of booster* session on student outcomes in grade 8** | | | | | | | | | | | | |
| --- | --- | --- | --- | --- | --- | --- | --- | --- | --- | --- | --- | --- |
| Variables | Estimated models | | | | | | | | | | | |
|  | HIV/AIDS knowledge | | | Preventive reproductive health skills | | | Self-efficacy | | | Intention to use protection | | |
|  | Β | SE | *t* | β | SE | t | β | SE | t | β | SE | *t* |
| *Fixed effect* |  |  |  |  |  |  |  |  |  |  |  |  |
| Intercept | 9.903 | 0.345 | 28.68^***^ | 4.347 | 0.338 | 12.86^***^ | 1.924 | 0.155 | 12.45^***^ | 4.643 | 0.225 | 20.65^***^ |
| Age | 0.011 | 0.025 | 0.42 | 0.004 | 0.029 | 0.13 | -0.015 | 0.013 | -1.14 | -0.058 | 0.017 | -3.32^***^ |
| Gender |  |  |  |  |  |  |  |  |  |  |  |  |
| Male | 0.031 | 0.084 | 0.37 | 0.027 | 0.045 | 0.59 | 0.017 | 0.044 | 0.39 | 0.103 | 0.061 | 1.68^#^ |
| Female (ref) |  |  |  |  |  |  |  |  |  |  |  |  |
| Baseline student outcome | 0.031 | 0.017 | 1.82^#^ | 0.037 | 0.017 | 2.14^*^ | 0.015 | 0.021 | 0.71 | 0.035 | 0.018 | 1.94^*^ |
| Grade 7 teacher’s level of  implementation of booster session |  |  |  |  |  |  |  |  |  |  |  |  |
| 4~5 activities completed | 0.496 | 0.203 | 2.44^*^ | -0.028 | 0.100 | -0.28 | 0.206 | 0.086 | 2.40^*^ | -0.045 | 0.142 | -0.31 |
| 2~3 activities completed | 0.513 | 0.190 | 2.70^**^ | -0.036 | 0.094 | -0.39 | 0.207 | 0.082 | 2.53^*^ | -0.016 | 0.134 | -0.12 |
| 0~1 activities completed (ref) |  |  |  |  |  |  |  |  |  |  |  |  |
| *Random effect* |  |  |  |  |  |  |  |  |  |  |  |  |
| School^†^ | 0.036 | 0.052 | 0.70 | 0.013 | 0.010 | 1.37^#^ | - | - |  | 0.006 | 0.020 | 0.30 |
| Class (nested within school)^†^ | 0.340 | 0.082 | 4.15^***^ | 0.015 | 0.012 | 1.24 | - | - |  | 0.138 | 0.037 | 3.71^***^ |
| # P<0.10; * P<0.05; ** P<0.01; *** P<0.001. ^†^ z test. | | | | | | | | | | | | |
