# Supplementary material for: Multi-year school-based implementation and student outcomes of an evidence-based risk reduction intervention
Source: Implement Sci. 2017 Feb 10;12:16. doi: 10.1186/s13012-016-0539-7 (PMC5303204; doi:10.1186/s13012-016-0539-7)
Supplement: Additional file 3: — Mixed-effects models assessing the effects of grade 8 teacher’s delivery of booster session on student outcomes in grade 8. (DOC 52 kb) [file 13012_2016_539_MOESM3_ESM.doc]

| **Additional file 3 Mixed-effects models assessing the effects of grade *8 teacher’s delivery of booster session* on student outcomes in grade 8** | | | | | | | | | | | | |
| --- | --- | --- | --- | --- | --- | --- | --- | --- | --- | --- | --- | --- |
| Variables | Estimated models | | | | | | | | | | | |
|  | HIV/AIDS knowledge | | | Preventive reproductive health skills | | | Self-efficacy | | | Intention to use protection | | |
|  | Β | SE | *t* | β | SE | t | β | SE | t | β | SE | *t* |
| *Fixed effect* |  |  |  |  |  |  |  |  |  |  |  |  |
| Intercept | 10.144 | 0.321 | 31.57^***^ | 4.377 | 0.160 | 27.28^***^ | 1.959 | 0.148 | 13.27^***^ | 4.288 | 0.208 | 20.62^***^ |
| Age | 0.017 | 0.025 | 0.71 | -0.010 | 0.013 | -0.82 | -0.015 | 0.013 | -1.23 | -0.058 | 0.017 | -3.37^***^ |
| Gender |  |  |  |  |  |  |  |  |  |  |  |  |
| Male | 0.009 | 0.081 | 0.11 | 0.010 | 0.043 | 0.24 | 0.034 | 0.043 | 0.79 | 0.089 | 0.059 | 1.51 |
| Female (ref) |  |  |  |  |  |  |  |  |  |  |  |  |
| Baseline student outcome | 0.025 | 0.016 | 1.55 | 0.027 | 0.017 | 1.62^#^ | 0.013 | 0.020 | 0.62 | 0.036 | 0.017 | 2.12^*^ |
| Grade 8 teacher’s level of  implementation of booster session |  |  |  |  |  |  |  |  |  |  |  |  |
| 4~5 activities completed | 0.258 | 0.151 | 1.71^#^ | 0.244 | 0.079 | 3.11^**^ | 0.215 | 0.073 | 2.95^**^ | 0.354 | 0.107 | 3.30^***^ |
| 2~3 activities completed | 0.135 | 0.155 | 0.87 | 0.020 | 0.081 | 0.25 | 0.126 | 0.076 | 1.67^#^ | 0.364 | 0.110 | 3.30^***^ |
| 0~1 activities completed (ref) |  |  |  |  |  |  |  |  |  |  |  |  |
| *Random effect* |  |  |  |  |  |  |  |  |  |  |  |  |
| School^†^ | 0.010 | 0.038 | 0.25 | 0.010 | 0.008 | 1.29 | - | - |  | 0.007 | 0.018 | 0.42 |
| Class (nested within school)^†^ | 0.344 | 0.077 | 4.50^***^ | 0.018 | 0.012 | 1.51^#^ | 0.006 | 0.009 | 0.69 | 0.132 | 0.035 | 3.83^***^ |
| # P<0.10; * P<0.05; ** P<0.01; *** P<0.001. ^†^ z test. | | | | | | | | | | | | |
